# Manometrically jackhammer esophagus with fluoroscopically/endoscopically distal esophageal spasm: a case report

**DOI:** 10.1186/s12876-021-01808-3

**Published:** 2021-05-17

**Authors:** Apichet Sirinawasatien, Pallop Sakulthongthawin

**Affiliations:** Division of Gastroenterology, Department of Medicine, Rajavithi Hospital, College of Medicine, Rangsit University, 2, Phayathai Road, Rajathewi, Bangkok, 10400 Thailand

**Keywords:** Jackhammer esophagus, Corkscrew esophagus, High-resolution manometry, Dysphagia, Case report

## Abstract

**Background:**

Jackhammer esophagus is a rare esophageal motility disorder that can result in dysphagia, chest pain, and gastro-esophageal reflux symptoms. High-resolution manometry is the gold standard for diagnosis, while corkscrew esophagus on upper gastrointestinal endoscopy is an uncommon manifestation.

**Case presentation:**

72-year-old man who presented with progressive dysphagia for three months without symptoms of chest pain or heartburn. Initial workup showed a corkscrew esophagus on upper gastrointestinal endoscopy; subsequently, high-resolution manometry revealed an esophago-gastric junction outflow obstruction with hypercontractile (jackhammer) esophagus. Treatment with calcium channel blockers and proton pump inhibitors was successful and relieved his symptoms near completion.

**Conclusions:**

Even though the corkscrew esophagus is typically for distal esophageal spasm, the hypercontractile (jackhammer) esophagus can appear. The high-resolution manometry can help to distinguish each specific motility disorder.

## Background

Jackhammer (JH) esophagus is defined as normally propagated esophageal peristaltic contractions with extremely elevated amplitudes [1]. Patients with this esophageal hypercontractility disorder normally present with dysphagia and noncardiac chest pain. A corkscrew appearance in barium studies and upper gastrointestinal (GI) endoscopy has been described in distal (or also called diffuse) esophageal spasm [2, 3], while in JH esophagus it is an uncommon finding. Nowadays, high-resolution manometry (HRM) is the gold standard for the diagnosis of this esophageal motility disorder [4]. Recently, the new Chicago classification version 4.0 describes the diagnostic criteria for JH esophagus as at least 20 % of swallows with a distal contractile integral (DCI) of over 8000 mmhg-s-cm on esophageal HRM [5]. Here, we reported a rare case of manometrically JH esophagus with fluoroscopically/endoscopically distal esophageal spasm.

## Case presentation

A 72-year-old man presented with three months’ history of progressive dysphagia for solid and liquid foods. He also had frequent instances of regurgitation of food without any symptoms of chest pain or heartburn, while his body weight remained constant. His Eckard score was 5 points which consist of dysphagia each meal and daily regurgitation. He had underlying hypertension and dyslipidemia and was taking losartan 50 mg/day and simvastatin 20 mg/day. He denied any history of smoking, alcohol drinking, or family history of cancer. Physical examination and basic laboratory tests were within normal limits.

**Fig. 1:**
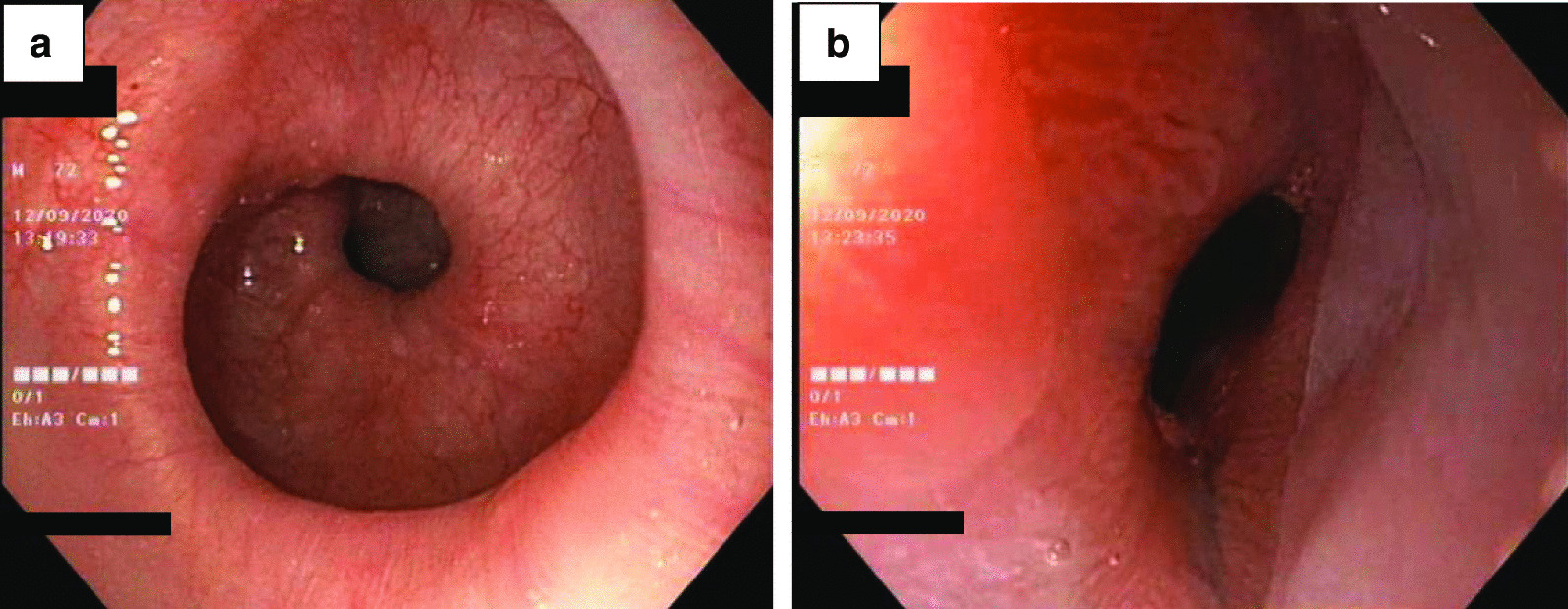
Upper gastrointestinal endoscopy revealed the corkscrew appearance of the lower esophagus (**a**); and normal esophagogastric junction (**b**)

**Fig. 2:**
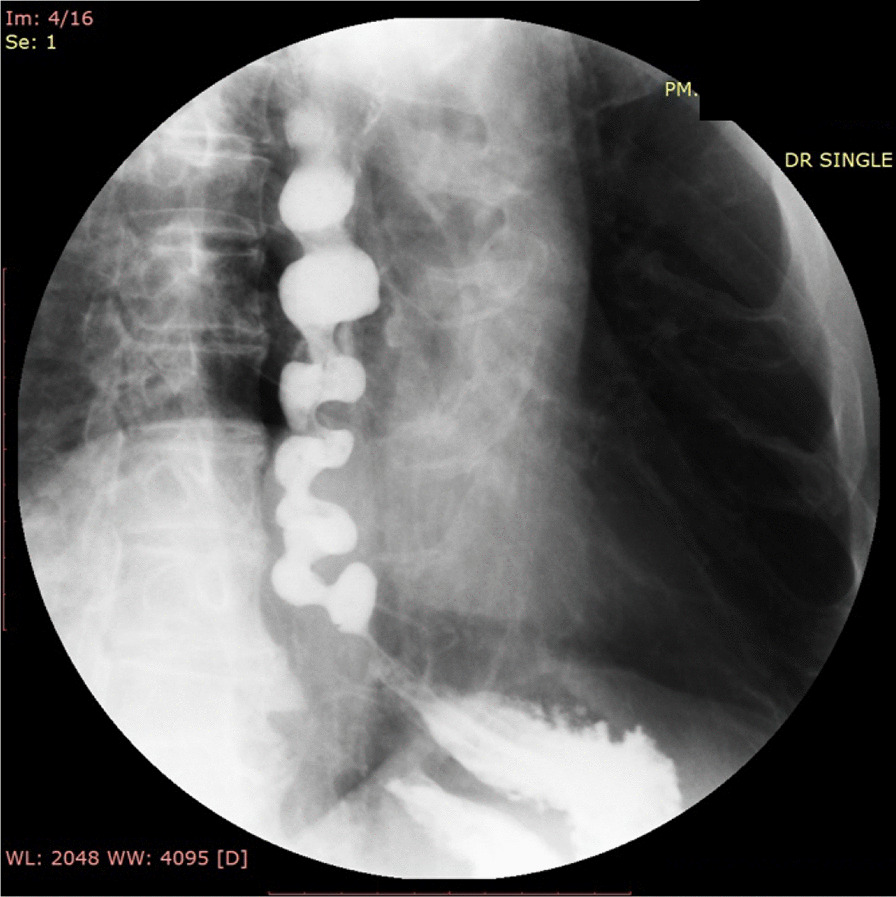
Barium esophagogram showed a constricted and curling of the esophageal lumen resembling a corkscrew

**Fig. 3:**
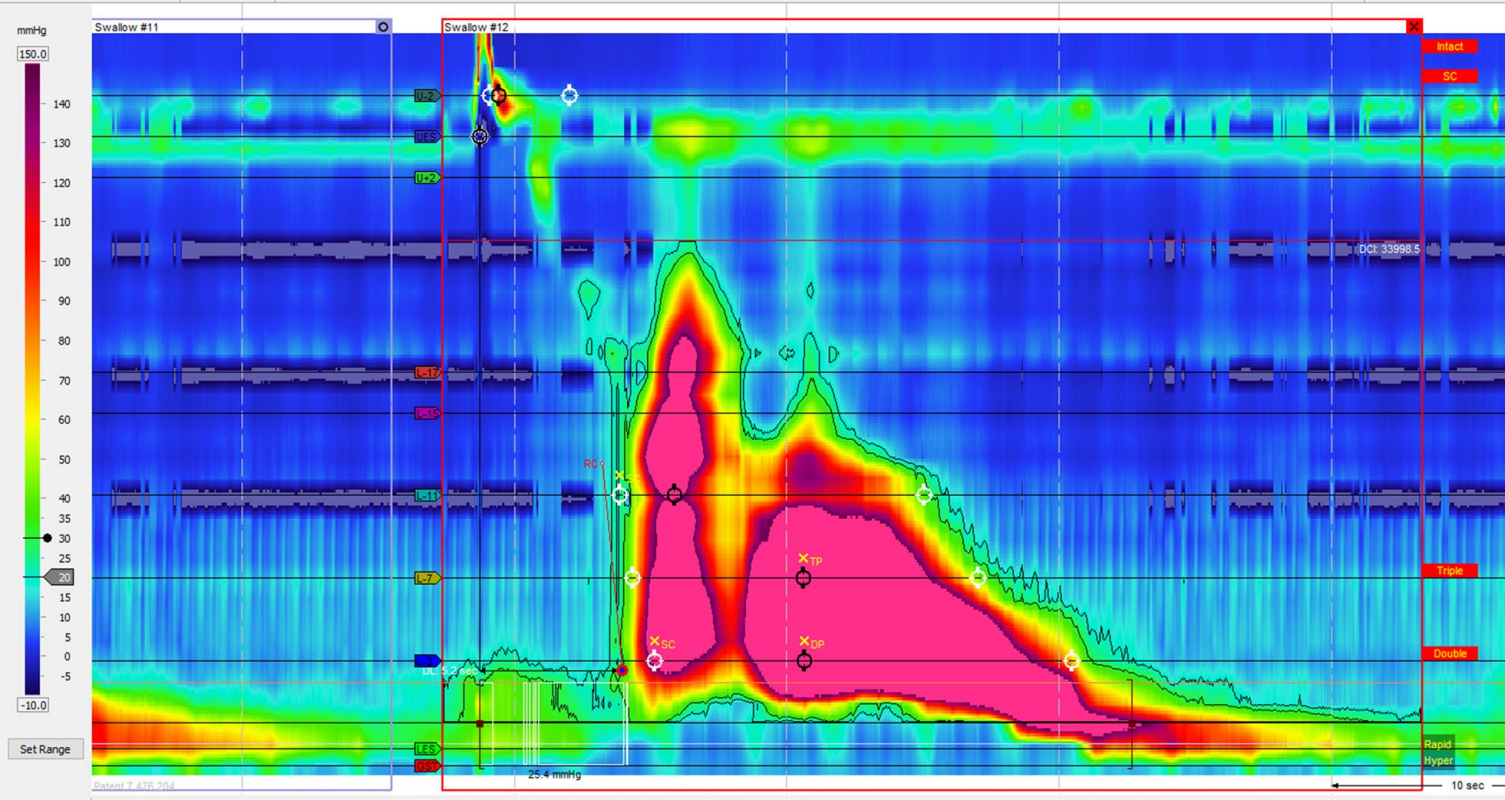
High-resolution esophageal manometry representative of the patient’s swallows. The median integrated relaxation pressure (IRP) was borderline high at 15.5 mmHg, a distal latency (DL) was normal at 6.5 s and the mean distal contractile integral (DCI) was elevated to 14,458 mmHg-s-cm. High amplitude peristaltic esophageal contraction with a DCI > 8000 mmHg-s-cm represented the hypercontractile (JH) esophagus

Upper GI endoscopy demonstrated circular folds like a winding staircase in the lower esophagus, which is classically known as a corkscrew esophagus (Fig. 1a), and a normal esophagogastric junction (Fig. 1b). A barium esophagogram showed constriction and curling of the esophageal lumen resembling a corkscrew (Fig. 2). He underwent HRM which showed 6 of 10 swallows with a DCI > 8000 mmhg-s-cm (the median with interquartile range of DCI was 16,597 [8736–30,397] mmhg-s-cm), a distal latency (DL) was normal at 6.5 s (the median with interquartile range of DL was 5.4 [3.3–5.7] s), and integrated relaxation pressure (IRP) was borderline high at 15.5 mmHg (Fig. 3). During the procedure, we observed quite good lower esophageal sphincter relaxation during wet swallowing.

Based on this HRM study, we made a diagnosis of an esophago-gastric junction (EGJ) outflow obstruction with hypercontractile (JH) esophagus. The patient was treated with a calcium blocker and proton pump inhibitor for one month after which his symptoms markedly reduced. After the treatment, the Eckard scores declined from 5 to 1, consisting of occasional dysphagia.

## Discussion and conclusions

JH esophagus is an uncommon esophageal motility disorder identified in 4 % of patients who undergo manometry [1]. It is an extreme phenotype of the hypercontractility esophageal disorder, described less than a decade ago, which is characteristics repetitive and excessively forceful contractions during peristalsis [6]. This disorder preserved normal contractile propagation and normal distal contractile latency, thereby excluding achalasia and distal esophageal spasm [6]. Hypercontractile (JH) esophagus can be a manifestation of EGJ outflow obstruction as evident by instances in which it occurs in association with an IRP greater than the upper limit of normal. This has several potential etiologies, including incompletely expressed achalasia or early achalasia. Supporting this contention, among a series of 12 patients diagnosed with JH esophagus on HRM, 3 (25 %) progressed to type III achalasia, 1 (8 %) reverted to normal manometry, and 8 (67 %) had persistent JH esophagus over a mean of 24 months. Whereas, a manometric finding of impaired EGJ relaxation found at the time of diagnosis with JH esophagus may predict this progression. [7]

These patients have major symptoms of dysphagia (72 %), pyrosis (42 %), retrosternal chest pain (36 %), and epigastralgia (33 %) [4]. Dysphagia is associated with strong contractions of the LES reflex, possible outflow obstruction, and a very high DCI, whereas chest pain is not associated with any of the manometric findings [8].

Various endoscopic findings have been reported in patients with JH esophagus. Mallet et al. reported that 54.3 % of these patients had gastro-esophageal reflux disease (GERD), while an upper GI endoscopy showed non-erosive to mild reflux esophagitis (Los Angeles grade A) [9]. A case report by Tanaka et al. revealed abnormally strong contractions in the distal esophagus on upper GI endoscopy and pathological results of the esophageal biopsy specimens showed massive eosinophil infiltration into the epithelium, which were diagnosed as JH esophagus due to eosinophilic esophagitis [10]. In another study by Clément et al. found that most of them were normal, about one-third who had abnormal findings during endoscopy included hiatal hernia, longitudinal striae, impression of esophageal dilatations, and increased LES tone. [4]

Overall, no typical endoscopic feature which was a hallmark for JH esophagus, while a corkscrew esophagus was an uncommon finding on upper GI endoscopy reports in literatures such as the one in our present study. Meanwhile, a typical corkscrew appearance of the lower esophagus is usually associated with distal esophageal spasm [2, 3], however, similar endoscopic findings to corkscrew esophagus in a patient with dysphagia was reported by Han and Wagh, but that patient was finally diagnosed with type III (spastic) achalasia by HRM [11].

Currently, the standard treatment of this disease is not well defined. Pharmacologic treatment should be selected as the first choice, includes smooth muscle relaxants such as calcium channel blockers, nitrates, or phosphodiesterase-5 inhibitors, which reduce both LES pressure and esophageal contraction amplitude [10]. Another option which can relieve chest pain symptoms without improvement in motility function is low-dose antidepressants [12]. Endoscopic therapies for refractory symptoms which aim to decrease the abnormal contraction vigor include botulinum toxin injection, pneumatic dilation, and peroral endoscopic myotomy all of which have been reported to achieve some success [13–15].

The rational of proton pump inhibitor used in this patient based on the pathophysiological mechanisms that esophageal acid perfusion can induce esophageal spasm [16], as well as, there was some evidence showed that spastic motility disorders can also occur in conjunction with, or as a consequence of GERD [6, 9, 17, 18]. Nevertheless, the flaw of the present report was the lack of the evidence of GERD by either pH-testing or endoscopy.

In summary, we describe a rare case of JH esophagus with a corkscrew esophagus on upper GI endoscopy, the classic finding for which an endoscopist is usually diagnosed with distal esophageal spasm; however, other possible diagnoses such as achalasia should be kept in mind before the disease has been confirmed by HRM.

## Data Availability

The data used to support the findings of this case report are included within the article.

## Abbreviations

JH: Jackhammer
GI: Gastrointestinal
HRM: High-resolution manometry
DCI: Distal contractile integral
DL: Distal latency
IRP: Integrated relaxation pressure
LES: Lower esophageal sphincter
GERD: Gastro-esophageal reflux disease
